# Rapid Desensitization to Reslizumab in an 80-Year-Old Woman With Chronic Eosinophilic Pneumonia, Severe Persistent Asthma, and Chronic Urticaria

**DOI:** 10.7759/cureus.96590

**Published:** 2025-11-11

**Authors:** Vicken Khazar, Samuel Escobar, James Kim, Steve McClain, Anthony M Szema

**Affiliations:** 1 Medicine, Donald and Barbara Zucker School of Medicine at Hofstra/Northwell, Hempstead, USA; 2 Medicine, Warren Alpert Medical School at Brown University, Providence, USA; 3 Pathology, McClain Laboratories LLC, Smithtown, USA; 4 Allergy and Immunology, Pulmonary Disease, Donald and Barbara Zucker School of Medicine at Hofstra/Northwell, Hempstead, USA

**Keywords:** drug-related side effect, idiopathic chronic eosinophilic pneumonia, pulmonary function test, rapid desensitization, reslizumab, severe persistent asthma

## Abstract

Our patient is an 80-year-old woman with chronic eosinophilic pneumonia (CEP), chronic urticaria (CU), severe persistent asthma (SPA), long-term oral corticosteroid therapy, and a resected papillary thyroid carcinoma. The patient had normal, stable immunoglobulin E (IgE) levels across all treatments ranging from 16 to 37 IU/mL. In treating her CEP and severe asthma, she trialed monoclonal antibody therapy with mepolizumab, benralizumab, dupilumab, and omalizumab, which were each begun and then discontinued successively due to severe side effects or allergic reaction. However, reslizumab offered 10 months of notable relief from urticaria and dyspnea without oral corticosteroids but was discontinued after the patient developed infusion-associated urticaria.

Given her relief with reslizumab, we planned to desensitize her to this medication via a stepwise, slowed infusion up to a target therapeutic dose. Complete vital signs and physical exam looking for symptoms of an allergic response, including angioedema, urticaria, dyspnea, and nausea, were performed every 15 minutes.

Our patient was successfully desensitized to reslizumab at a target dose of 195 mg over three hours. Vitals were checked every 15 minutes, which were within normal limits throughout. Further, she was monitored for any symptoms of allergic reaction, including angioedema, nausea, urticaria, and dyspnea, and denied any complaints throughout the procedure. Our patient reported notable relief of dyspnea and fatigue at two weeks and four weeks post-procedure.

As opposed to a decrease in dose or discontinuation of treatment, patients may undergo rapid desensitization through a stepwise, slowed infusion rate to better tolerate a previously allergenic medication.

## Introduction

Chronic eosinophilic pneumonia (CEP) is a rare disorder characterized by an accumulation of eosinophils in the interstitium and alveolar spaces of the lungs. Symptoms manifest gradually and commonly include fever, weight loss, dyspnea, productive asthma, and night sweats [1]. The diagnosis of CEP is made radiologically with chest radiograph or CT chest showing peripheral airspace consolidations (also known as reverse batwing opacities), while lung biopsy shows eosinophilic pulmonary infiltrates.

Chronic urticaria (CU) is characterized by recurrent pruritic maculopapular wheals, angioedema, or both for greater than six weeks. Diagnosis of CU is marked by a CU index score greater than 10, which measures the amount of histamine released from donor basophils when mixed with the patient's serum. Urticaria occurs due to mast cell and basophil activation and subsequent rupture of granules containing histamine, bradykinin, and kallikrein. These molecules cause vasodilation, increased vascular permeability, and ultimately and intradermal edema and erythema [2]. CU is generally self-limiting, with symptoms lasting two to five years on average.

Severe persistent asthma is a chronic inflammatory respiratory condition with varying presentations, severities, and diagnostic results. It is characterized by inflammation of the airways, causing persistent airflow obstruction and bronchial hyperresponsiveness, which worsens during acute exacerbation by various triggers such as allergens or infections. Hallmark symptoms include coughing, wheezing, and shortness of breath. Severe persistent asthma is often refractory to standard asthma treatments such as long-acting beta agonists (LABAs) and long-acting muscarinic antagonists (LAMAs).

Reslizumab is an interleukin-5 (IL-5) antagonist monoclonal antibody therapy, approved by the Food and Drug Administration (FDA) for adults with severe asthma with eosinophilic phenotype, which is a result of eosinophil proliferation [3]. This drug binds to the IL-5 alpha chain receptor on eosinophils and inhibits their proliferation. However, reslizumab is known to cause allergic reactions (including symptoms of angioedema, asthma exacerbations, and urticaria), which are often severe enough to warrant discontinuation of the drug [4]. Thus, for patients who have clinical improvement with reslizumab but develop an allergic reaction, rapid desensitization to this drug may be the potential solution.

## Case presentation

**Figure 1:**
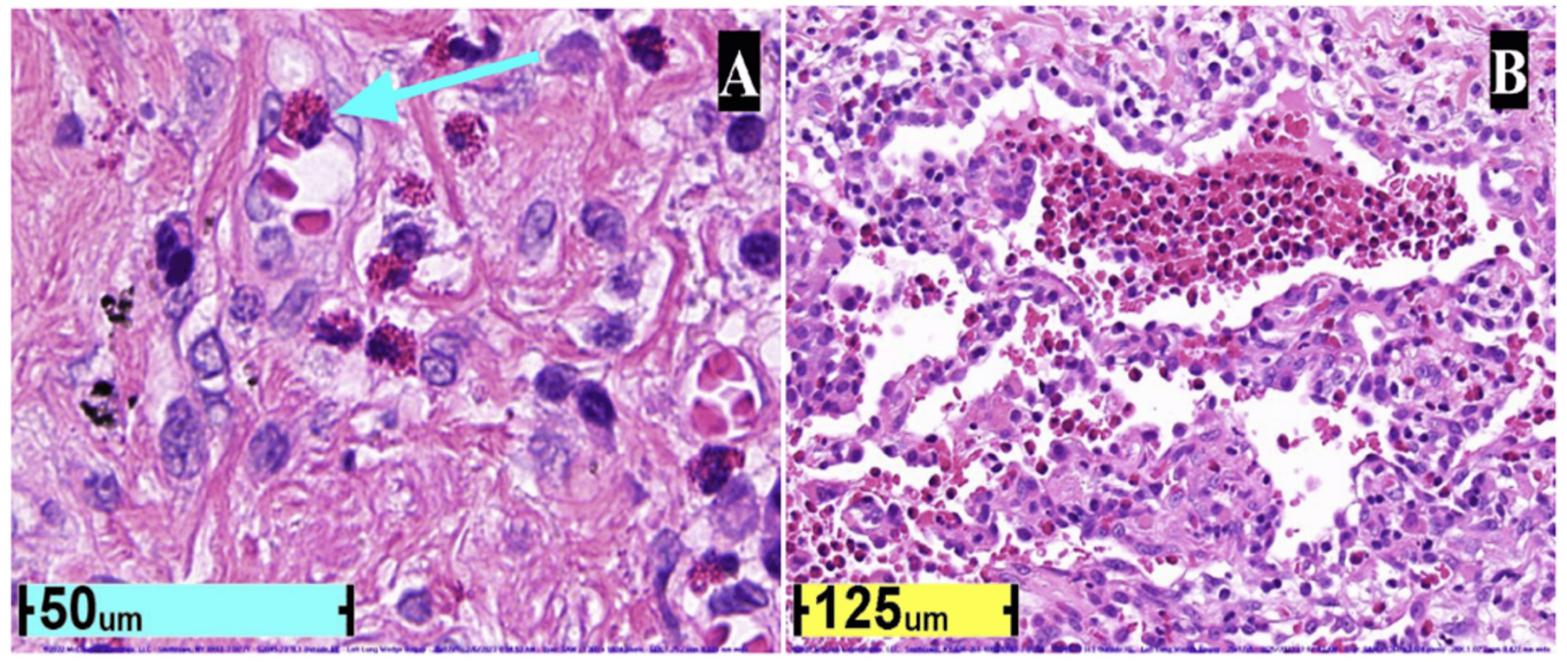
Histopathology (hematoxylin & eosin stain) of transbronchial left lung biopsy. Images reveal marked infiltration of eosinophils at 800x (A) and 400x (B) magnification and BOOP-like activation of exudates. Eosinophils are characterized by bilobed nuclei and red stained cytoplasm (teal arrow). BOOP: Bronchiolitis obliterans organizing pneumonia.

We present an 80-year-old never-smoker Caucasian woman with a past medical history of resected papillary thyroid carcinoma diagnosed with CEP, severe persistent asthma with concomitant severe urticaria, arthralgia, and fibrous dysplasia of bone. The patient presented in the clinic with dyspnea, weight loss, urticaria, and productive cough. Her lung biopsy showed infiltration of eosinophils and bronchiolitis obliterans organizing pneumonia (BOOP)-like activation of exudates (or cryptogenic organizing pneumonia), which is consistent with CEP. The lung biopsy was negative for necrosis, granuloma formation, mucous-plugging of bronchi, or bronchocentric pathology (Figure 1).

**Figure 2:**
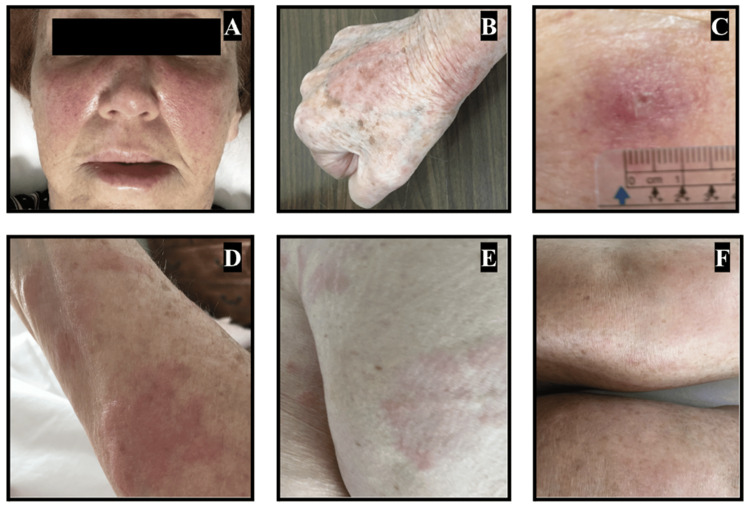
Maculopapular, pruritic, erythematous urticaria to the (A) face, (B) dorsal right hand and wrist, (C) right lateral forearm, (D) left lateral forearm, (E) right medial upper thigh, and (F) bilateral anterior knees. Images were taken one day after the tenth infusion of reslizumab.

**Figure 3:**
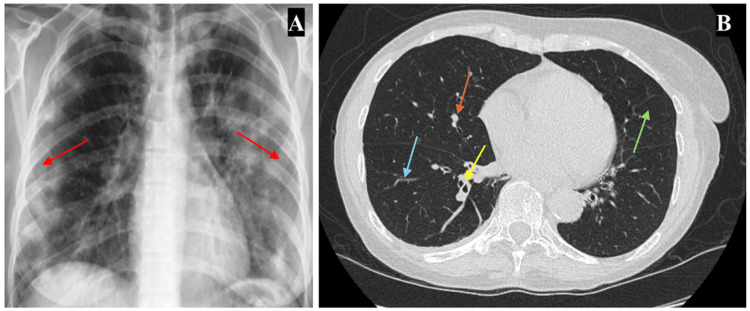
(A) Chest X-ray shows peripheral airspace opacities sparing the perihilar region (reverse batwing sign), as indicated by red arrows. (B) Chest CT shows small pulmonary nodules (orange arrow), vascular walls with mural enhancement (light blue arrow), persistent lower lobe bronchial wall thickening (yellow arrow), and mild emphysema (green arrow).

Her urticarial flair-ups were most often distributed on the face, trunk, and bilateral arms, hands, and legs (Figure 2). Chest X-ray showed peripheral airspace opacities (reverse batwing sign) sparing the perihilar region (Figure 3A), while chest CT showed small pulmonary nodules, mural enhancement of vascular walls, persistent lower lobe bronchial wall thickening, and mild emphysema (Figure 3B). Further, she was diagnosed with CU at age 65 with a CU index of 50. Total IgE levels were within normal limits throughout all treatments (16-37 IU/mL). Similarly, serum tryptase was normal at 8.3 ng/L and a complete blood count with differential showed a white blood cell count of 3.41 K/μl with an absolute eosinophil count of 0.04, making a diagnosis of hypereosinophilic syndrome very unlikely.

The patient underwent extensive pulmonary function testing prior to beginning reslizumab infusions. Lung volumes, impulse oscillometry (IOS), and diffusion capacity were normal throughout. Spirometry showed mild airway obstruction and small airways dysfunction. Exhaled nitric oxide (NiO*_x_*) was elevated at 55 ppb (Table 1).

**Table 1:** Pulmonary function testing with spirometry, exhaled NiOx, IOS, lung volumes, diffusion, and airways resistance prior to using reslizumab. IOS: Impulse oscillometry FVC: Forced vital capacity FEV1: Forced expiratory volume in 1 second FEF: Forced expiratory flow FIF Max: Forced inspiratory flow, maximum MVV: Maximal voluntary ventilation MEP: Maximal expiratory pressure MIP: Maximal inspiratory pressure FEF Max: Forced expiratory flow, maximum R5Hz: Resistance to airflow measured at a frequency of 5 Hz R20Hz: Resistance to airflow measured at a frequency of 20 Hz X5Hz: Reactance of peripheral airways at 5 Hz SVC: Slow vital capacity IC: Inspiratory capacity ERV: Expiratory reserve volume TGV: Thoracic gas volume RV: Residual volume TLC: Total lung capacity Raw: Airway resistance Gaw: Airway conductance sRaw: Specific airway resistance sGaw: Specific airway conductance DLCOunc: Diffusing capacity for carbon monoxide uncorrected for hemoglobin Kco: Transfer coefficient (diffusing capacity corrected for alveolar volume) DL: Diffusing lung capacity VA: Alveolar volume

Pulmonary Function Testing	Predicted value	Actual value	Percent of predicted value (%)
Spirometry			
FVC (L)	2.65	2.30	87
FEV1 (L)	1.98	1.88	94
FEV1/FVC (%)	77	64	83
FEF 25%-75% (L/sec)	1.61	0.85	52
FIF Max (L/s)	N/A	3.08	N/A
MVV (L/min)	81	68	83
MEP (cm H_2_O)	121	44	36
MIP (cm H_2_O)	-60	-44	72
FEF Max (L/s)	4.98	5.28	106
Impulse oscillometry			
R5Hz	4.31	2.49	58
R20Hz	3.7	2.15	58
X5Hz	-1.25	-1.01	81
Lung volumes			
SVC (L)	2.60	3.23	124
IC (L)	1.78	2.15	121
ERV (L)	0.89	1.07	120
TGV (L)	2.91	3.74	128
RV (L)	2.35	2.67	113
TLC (L)	5.05	5.89	116
RV/TLC	46.80	45.27	96
Exhaled nitric oxide			
NiOx	<12	55	N/A
Airway resistance			
Raw (cmH20/L/s)	1.86	1.49	80
Gaw (L/s/cmH2O)	1.03	0.68	65
sRaw (cm H_2_O*s)	<4.76	5.57	N/A
sGaw (l/cm H_2_O*s)	0.20	0.18	88
Diffusion			
DLCOunc (ml/min/mmHg)	18.92	15.21	80
DL/VA (ml/min/mmHg/L)	4.08	3.29	80
VA (L)	4.63	4.62	99

Our patient was treated with oral corticosteroids for the past 18 years and developed severe osteoporosis. She began treatment of CEP and CU with mepolizumab, dupilumab, and then benralizumab, each with mild relief of dyspnea and urticaria, but each medication was stopped after two months when she developed vomiting, angioedema, and arthralgias. She then began omalizumab, which she stopped after one dose after developing hyperacute tachycardia and myalgias. Finally, she began on reslizumab infusions, which offered complete relief of dyspnea and urticaria while stopping oral corticosteroids for 10 months. However, reslizumab was stopped at the 10th month when she experienced multiple post-infusion urticarias. She presented to the allergist/immunologist with urticaria on her face, bilateral forearms, legs, and upper chest. The rash was self-limiting over one week post-infusion but became more severe and lasted for a longer duration after each subsequent infusion. She then restarted reslizumab after a three-month-long hiatus, but had recurrent hives on rechallenge within 30 minutes. Further, the rash was unresponsive to treatment with loratadine, cetirizine, fexofenadine, or triamcinolone topical ointment. Table 2 shows the patient’s complete treatment history, including degree of relief, duration, and side effect(s).

**Table 2:** History of treatments with subjective degree of relief, duration, and limiting side effects.

Treatment	Relief of Dyspnea	Relief of Urticaria	Reason for Discontinuation
Prednisone	Mild	Mild	Osteoporosis after 18 years
Mepolizumab	Mild	Mild	Joint pain and myalgias after 2 months
Benralizumab	Mild	Mild	Joint pain and vomiting after 2 months
Omalizumab	None	None	Myalgias and tachycardia after 1 week
Dupilumab	Mild	Mild	Vomiting and angioedema after 2 months
Reslizumab	Resolved	Resolved	Severe urticaria and angioedema after 10 months

Given the patient’s months-long success with reslizumab, as compared to other treatments, and history of infusion-related reactions, we conducted a desensitization protocol to reslizumab. Desensitization is a stepwise titration and administration of a drug through a series of diluted concentrations until a tolerated target dose is reached. A five-step protocol was used based on the existing protocols of desensitization to omalizumab. Vitals were checked every 15 minutes and were within normal limits throughout. Further, she was monitored for symptoms of allergic reaction, including angioedema, nausea, urticaria, and dyspnea. Our patient reported notable relief of dyspnea and fatigue two weeks post-desensitization.

Table 3 shows the five-step protocol used. The drug was administered over two hours up to a cumulative dose of 525 mg. Moving forward, our patient will continue receiving reslizumab at 195 mg monthly with continuous remote patient-monitoring and regular clinic visits.

**Table 3:** Reslizumab desensitization protocol with stepwise titration. Vitals were checked every 15 minutes, and the patient was monitored for 60 minutes post-procedure. A standard stock solution of 10 mg/mL of reslizumab was used for titration.

Infusion Step	Infusion Rate (mL/h)	Reslizumab Concentration (mg/mL)	Infusion Duration (min)	Volume to Infusion (mL)	Administered Dose (mg)	Cumulative Dose (mg)
1	10	10	15	2.5	25	25
2	20	10	15	5	50	75
3	40	10	15	10	100	175
4	60	10	15	15	150	325
5	80	10	15	20	200	525
Total time	180 minutes					

The patient returned to our clinic at two and four weeks post-procedure and reported zero instances of urticaria and notable relief of dyspnea without the use of oral corticosteroids. She also denied any symptoms of allergic reaction, including angioedema, urticaria, vomiting, tachycardia, and depressive episodes. The patient has been receiving reslizumab infusions monthly for the past three months with continued relief of dyspnea and urticaria. She also reports being able to exercise for the first time in the past 20 years without dyspnea.

## Discussion

While reslizumab demonstrates great success in treating asthmatic diseases with eosinophilic phenotype, adverse side effects such as urticaria and oropharyngeal pain are reported in 2.68% of patients [4]. The prescriber information recommends dosage reductions and temporary interruption of treatment in mild reaction cases, or permanent discontinuation for severe infusion reactions. However, such treatment modifications may not be suitable for patients with severe asthma complications and extensive past medical history, when the use of first-line therapies may be critical for symptom relief. Thus, patients may undergo desensitization through a stepwise, titrated infusion to better tolerate a target dose. Further, this case is unique in its novel treatment of both CEP and CU using reslizumab. Prior literature reports on the use of successful desensitization protocols to other monoclonal antibody therapeutics, such as omalizumab [5]. Nevertheless, this case report describes the first successful protocol for a desensitization to reslizumab in an 80-year-old woman with CEP, CU, and severe persistent asthma, which offers prescribers an alternative option to discontinuation or dose reduction in patients with allergic reactions.

This case shows how immune system dysregulation can manifest in multiple organs, so that Ockham's Razor ultimately prevails, but each individual diagnosis and side effect of treatment must be addressed.

## Conclusions

This case highlights the first successful desensitization to reslizumab in an elderly patient with chronic eosinophilic pneumonia, chronic urticaria, and severe persistent asthma. For patients who experience hypersensitivity reactions to otherwise effective biologic therapies, desensitization can provide a safe and practical alternative to drug discontinuation or dose reduction. Clinicians should consider desensitization protocols in select patients with limited therapeutic options, as this approach may restore disease control, reduce corticosteroid dependence, and improve quality of life.
